# Oxygen-dependent modulation of the human complement system during acute normobaric hypoxia: a translational plasma proteomics study

**DOI:** 10.1007/s10238-026-02084-9

**Published:** 2026-02-21

**Authors:** Alexander Lang, Tin Yau Pang, Sarah Piel, Daniel Oehler, Elric Zweck, Khatereh Shahrjerdi, Madlen Okkasian, Jacqueline Georgy, Yvonne Reinders, Ashley-Jane Duplessis, Jens Tank, Jens Jordan, Susanne Pfeiler, Albert Sickmann, Malte Kelm, Christian Jung, Norbert Gerdes

**Affiliations:** 1 Division of Cardiology, Pulmonology, and Vascular Medicine, Medical Faculty and University Hospital, Heinrich-Heine University, Moorenstr. 5, 40225 Düsseldorf, Germany; 2 Leibniz-Institut für Analytische Wissenschaften-ISAS-e.V, Dortmund, Germany; 3 Institute of Aerospace Medicine, German Aerospace Center (DLR), Cologne, Germany; 4 Medical Faculty, University of Cologne, Cologne, Germany; 5 Cardiovascular Research Institute Düsseldorf (CARID), Medical Faculty, Heinrich-Heine University, Düsseldorf, Germany; 6 Medical Proteom Center, Ruhr-University Bochum, Bochum, Germany; 7 Leibniz Institute for Diabetes Research, German Diabetes Center (DDZ), Düsseldorf, Germany

**Keywords:** Hypoxia, Reoxygenation, Complement system, Plasma proteomics, Innate immunity, Inflammation

## Abstract

Acute hypoxia triggers multiple physiological and immune responses, yet the immediate systemic effects on circulating complement proteins remain insufficiently characterized. The complement cascade plays a central role in inflammation, host defense, and ischemia-related tissue injury, but its regulation during transient oxygen deprivation and reoxygenation in humans is poorly understood. Sixteen healthy volunteers were exposed to stepwise normobaric hypoxia simulating altitudes of 0, 2, 4, and 6 km (pO₂ = 9.64 kPa) followed by reoxygenation under normoxic conditions. Blood samples were collected at baseline, peak hypoxia (6 km), and after reoxygenation. Quantitative plasma proteomics was performed using targeted multiple-reaction-monitoring mass spectrometry to quantify key complement components (C1 complex, C3–C9, factor B) in 16 participants with complete datasets. Hematological parameters were analyzed in parallel. Hypoxia transiently increased leukocyte and platelet count, whereas hematocrit and mean corpuscular volume slightly decreased. While only slightly increasing during hypoxia, most complement peptides - including C1S, C1R, C3, C5, C7, C9, and CFAB - showed a coordinated reduction in relative abundance upon reoxygenation compared to both baseline and hypoxia (median fold-change ≈ 0.6–0.8; *p* < 0.05). Correlation analysis revealed coherent clustering among complement components but only weak associations with hematological indices. Acute hypoxia elicits rapid and reversible changes in the circulating complement peptide pool in healthy humans. Targeted plasma proteomics demonstrates clear oxygen-phase–dependent dynamics, with a coordinated decrease after reoxygenation. This pattern is consistent with reduced circulating availability of complement components, activation-associated consumption, and/or redistribution within the intravascular compartment. Future validation of these findings in certain patient cohorts may define translational relevance and functional consequences.

**Graphical abstract:**

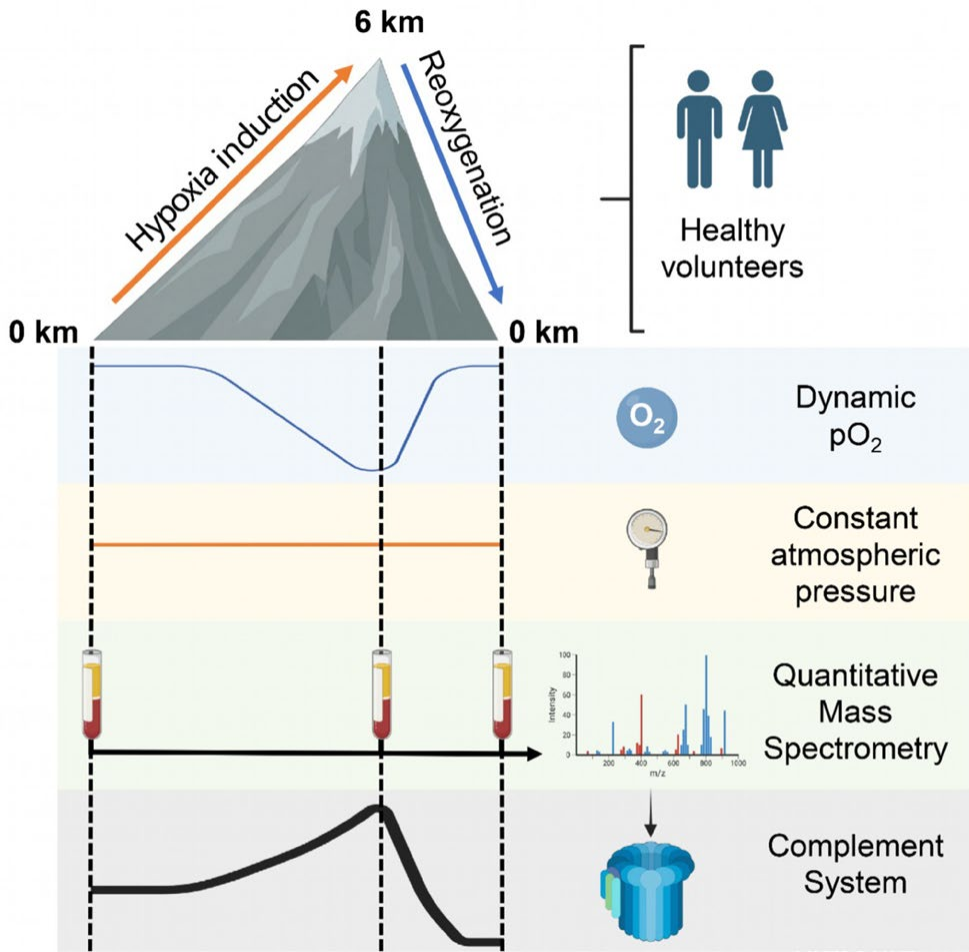
Sixteen healthy volunteers were exposed to stepwise normobaric hypoxia simulating altitudes of 0 km, 2 km, 4 km, and 6 km. Hypoxia increased plasma concentrations of several proteins of the complement system as analyzed by quantitative mass spectrometry. Upon reoxygenation, most complement peptides decreased to or below baseline.

## Introduction

Hypoxia, defined as a reduction in oxygen availability, is a common feature of many physiological and pathological conditions. It occurs in diverse contexts, ranging from high-altitude exposure to ischemic tissue injury, heart failure, and cardiogenic shock [1, 2]. Hypoxia influences not only cellular metabolism but also immune and inflammatory pathways through oxygen-sensitive signaling cascades such as hypoxia-inducible factors (HIFs) [3, 4]. Despite extensive work on chronic and tissue-specific hypoxia, the immediate systemic consequences of acute, reversible hypoxia in humans remain insufficiently characterized.

The complement system represents a key component of the innate immune response and contributes to inflammation, pathogen defense, and clearance of apoptotic cells [5]. Comprising more than 30 plasma proteins, the complement system can be activated through 3 major pathways (i.e., classical, lectin, and alternative) culminating in the assembly of the membrane attack complex (MAC) and the release of inflammatory and opsonizing mediators [6]. Dysregulated complement activation has been implicated in ischemia-reperfusion injury, cardiovascular disease, and metabolic disorders [7–9]. However, whether and how short-term systemic hypoxia and reoxygenation affect circulating complement proteins in humans is largely unknown.

Experimental data indicate that hypoxia can modulate complement component expression at the transcriptional level. In human macrophages, hypoxia increases C3 gene expression through HIF-1α–dependent mechanisms [10], whereas in endothelial cells, oxygen deprivation suppresses complement inhibitors such as CD55 and CD59 [11]. Recent proteomic studies of high-altitude exposure have shown transient plasma changes in C3 and C4 levels [12, 13], but these findings remain inconsistent and largely descriptive. A mechanistic link between oxygen deficit and systemic complement regulation is still undefined.

Acute normobaric hypoxia is a defining feature of myocardial infarction, cardiogenic shock, and organ ischemia, where reduced oxygen supply precedes the inflammatory burst that typically emerges during reperfusion. Complement activation is a major driver of this sterile inflammation, yet it remains unknown whether hypoxia alone already shifts circulating complement activity before tissue injury or reperfusion occur. This study examined whether oxygen deprivation by itself primes, suppresses, or otherwise alters complement regulation in humans. A controlled hypoxia model allowed isolation of the direct effect of reduced oxygen tension without confounding inflammation or necrosis. By combining targeted plasma proteomics with hematological profiling, the present work tested whether oxygen availability is associated with rapid changes in circulating complement proteins in vivo. This concept is relevant for early inflammatory events in ischemia reperfusion settings and clarifies whether hypoxia itself contributes to complement driven injury in cardiovascular disease.

## Materials and methods

### Ethics

The study was explorative in nature, approved by the ethics committee of the University Hospital of Heinrich-Heine-University, Düsseldorf, Germany (5925R) and conducted according to the principles of the Declaration of Helsinki and Good Clinical Practice. Informed consent was obtained from all subjects before enrollment.

### Study design

We recruited 16 healthy volunteers without cardiovascular disease. The study was conducted at the ‘DLR: envihab’ of the German Aerospace Center (https://www.dlr.de/envihab/), Cologne, Germany. Hypoxia was generated by progressively lowering the ambient oxygen fraction through controlled nitrogen enrichment while keeping temperature, humidity, atmospheric pressure, and pCO_2_ constant. Oxygen levels were adjusted stepwise to four predefined target pO_2_ plateaus corresponding to respective altitude above sea level: baseline 21.25 kPa (0 km), moderate hypoxia 16.42 kPa (2 km), marked hypoxia 12.63 kPa (4 km), and severe hypoxia 9.64 kPa (6 km). Volunteers remained at each oxygen level for two hours. The altitude labels (0, 2, 4, 6 km) served only as shorthand for the corresponding pO_2_ values. Blood was not collected at each step because the study focused on basal conditions, the maximal hypoxic load, and the immediate response to oxygen restoration. EDTA-anticoagulated whole blood was obtained at baseline, at the end of the 9.64 kPa exposure, and within 5–15 min after return to normoxia (reoxygenation). Sampling was performed within the final 5 min of each condition to ensure steady state. All volunteers completed the protocol during daytime hours in a single continuous session to minimize circadian variation. Plasma was isolated by centrifugation at 1000 x g for 10 min at room temperature, immediately aliquoted, and frozen at -80 °C until targeted proteomic analysis. The study was exploratory and the sample size was limited.

### Targeted plasma proteomics analysis

Plasma preparation was accomplished using the MRM Proteomics PeptiQuant™ Plus Kits (Cambridge Isotope Laboratories, Cambridge, MA, USA) according to the manufacturer’s instructions. MRM quantification reflects the abundance of selected proteotypic peptides and does not distinguish intact precursor proteins from proteolytic activation fragments, which is particularly relevant for complement components undergoing cleavage (e.g., C3/C5). Briefly, 10 µL plasma was denatured and reduced using 20 mM DTT in a 96-well plate. After alkylation using iodoacetamide, plasma was digested overnight at 37 °C using Sequencing Grade trypsin (Promega, Madison, WI, USA). Final protease:protein ratio was 1:20 (w/w). Subsequently, 2% formic acid (FA) was used to quench digestion, resulting in a total volume of 700 µL. Spike solution for standard curve and quality control was prepared and combined in the 96-well plate according to manufacturer instructions. Finally, samples were cleaned-up in 96-well format with solid phase extraction (SPE) using a Oasis HLB µ-elution plate (Waters, Milford, MA, USA) in a Resolvex^®^ A200 (TECAN, Männedorf, Switzerland). All samples were reconstituted in 0.1% FA and analyzed using HPLC-MS/MS. To that end, peptide samples were separated on an Ultimate 3000 Liquid chromatography system coupled to a TSQ Quantiva™ mass spectrometer (both Thermo Scientific, Hamburg, Germany). On the chromatographic system, peptides were trapped on a µ-Precolumn (Acclaim PepMap 100 C18, 2 μm, Thermo Scientific) and separated on a reverse phase main-column (Acclaim PepMap 100 C18, 300 μm x 5 mm, 5 μm, 100 A, Thermo Scientific) using a binary gradient consisted of A: 0.1% FA and B: 84% acetonitrile, 0.1% FA at a flow rate of 5 µl/min. The gradient increased linearly from 3% A to 50% B over 60 min. For the mass spectrometry analysis, the TSQ Quantiva measured after electrospray ionization with single reaction monitoring (SRM) in the positive ion mode using a spraying potential of 4000 V at least one transition per peptide. Resolution of Q1/Q3 was set to 0.7 full width at half-maximum (FWHM). A list of the selected quantified proteins of the complement system is shown in Table 1.

**Table 1:** Peptides quantified by mass spectrometry and corresponding complement proteins

Protein Description		Protein Accession	Name	Quantified Peptide
Complement C1q subcomponent subunit B OS=Homo sapiens GN=C1QB PE=1 SV=3		P02746	C1QB	IAFSATR
Complement C1q subcomponent subunit C OS=Homo sapiens GN=C1QC PE=1 SV=3		P02747	C1QC	FNAVLTNPQGDYDTSTGK
Complement C1r subcomponent OS=Homo sapiens GN=C1R PE=1 SV=2		P00736	C1R	GLTLHLK
Complement C1r subcomponent-like protein OS=Homo sapiens GN=C1RL PE=1 SV=2		Q9NZP8	C1RL	VVVHPDYR
Complement C1s subcomponent OS=Homo sapiens GN=C1S PE=1 SV=1		P09871	C1S	TNFDNDIALVR
Complement factor B OS=Homo sapiens GN=CFB PE=1 SV=2		P00751	CFAB	EELLPAQDIK
Complement C3 OS=Homo sapiens GN=C3 PE=1 SV=2		P01024	CO3	TGLQEVEVK
Complement C5 OS=Homo sapiens GN=C5 PE=1 SV=4		P01031	CO5	VFQFLEK
Complement component C7 OS=Homo sapiens GN=C7 PE=1 SV=2		P10643	CO7	LIDQYGTHYLQSGSLGGEYR
Complement component C9 OS=Homo sapiens GN=C9 PE=1 SV=2		P02748	CO9	LSPIYNLVPVK

Abbreviations: OS, original species; GN, gene name; PE, protein existence; SV, sequence version

### Statistical analysis

Statistical analysis was performed using GraphPad Prism (Version 10, Boston, MA, USA) and R (Version 4.1.2). Data are presented as median (IQR) unless stated otherwise. Repeated-measures comparisons across baseline, hypoxia, and reoxygenation were analyzed using repeated-measures one-way ANOVA with Tukey’s post-hoc test. For variables that did not meet normality assumptions, the Friedman test with Dunn’s correction was applied. Correlations were assessed using Pearson or Spearman correlation as appropriate. A two-sided p-value ≤ 0.05 was considered statistically significant.

## Results

### Study population and physiological parameters

A total of 16 healthy volunteers were included, of whom eleven were male (55%). The median age was 29 years (IQR 25–31), and the median body mass index (BMI) was 23 kg/m² (IQR 21–25.3). Baseline measurements showed a median systolic blood pressure (SBP) of 115 mmHg (IQR 106–128), a diastolic blood pressure (DBP) of 70 mmHg (IQR 64.5–75), a heart rate (HR) of 67 beats per minute (IQR 61.5–70), and a peripheral oxygen saturation (SpO₂) of 97% (IQR 96–98). Hypoxia was induced in a stepwise manner up to the equivalent of 6 km altitude (pO₂ = 9.64 kPa), with each level maintained for 2 h, followed by a reoxygenation phase at sea-level oxygen pressure. Sixteen participants completed the entire hypoxia protocol, grouped into 2 consecutive experimental runs. The partial pressure of carbon dioxide (pCO₂) remained stable throughout (median 0.046 kPa at 0 km, 0.060 kPa at 2 km, 0.072 kPa at 4 km, and 0.059 kPa at 6 km). Quantitative mass spectrometry analysis was successfully performed for 16 participants at all time points.

### Hematological changes under hypoxia and reoxygenation

**Fig. 1:**
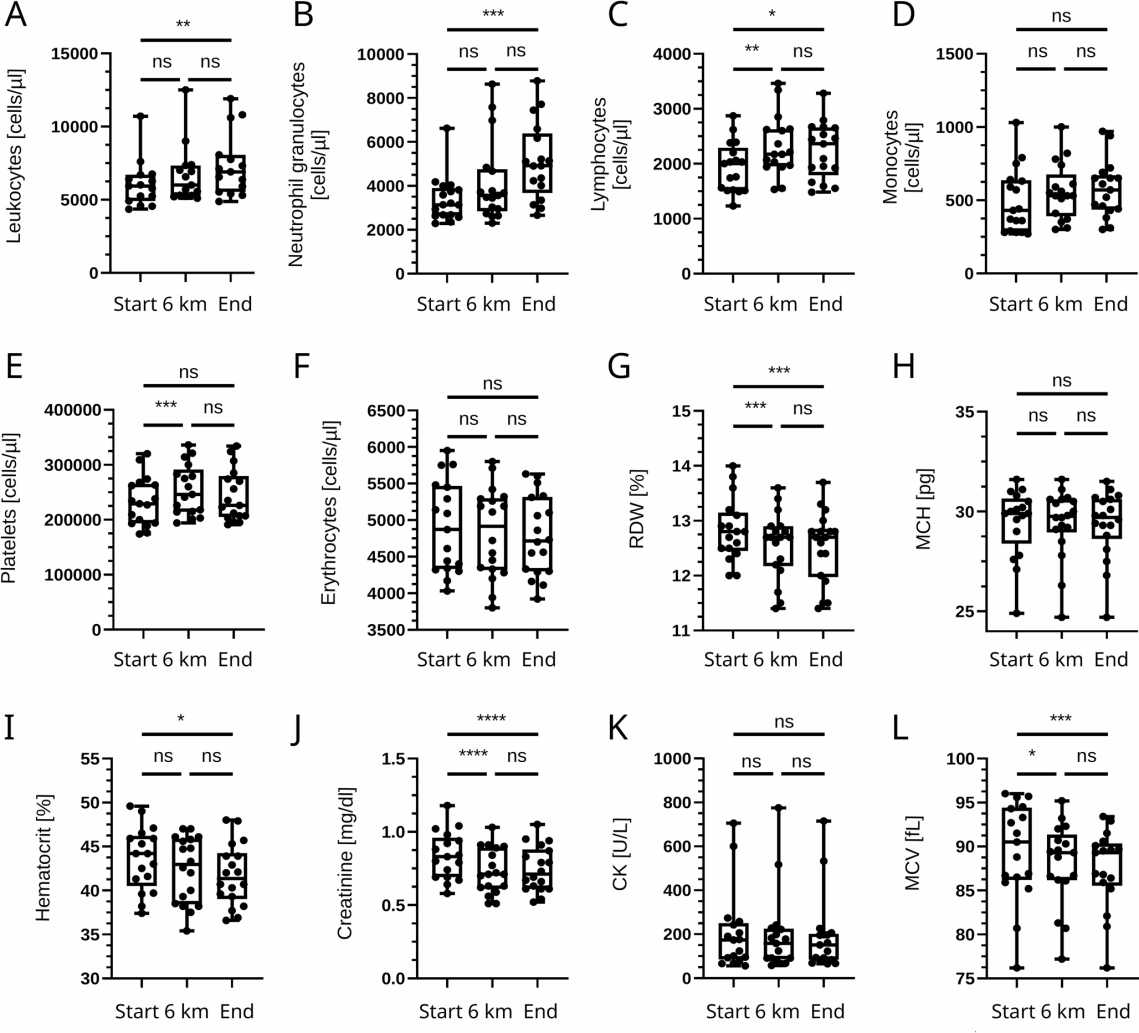
Hematological parameters under normobaric hypoxia and reoxygenation. Boxplots show circulating leukocytes, neutrophils, lymphocytes, monocytes, platelets, erythrocytes,  red-cell distribution width (RDW), mean corpuscular hemoglobin (MCH), hematocrit (HCT), creatinine, creatine kinase (CK), and mean corpuscular volume (MCV), mean corpuscular hemoglobin (MCH), and red-cell distribution width (RDW) measured at baseline (0 km), during hypoxia (6 km equivalent), and after reoxygenation. Individual data points represent values from each participant (*n* = 16). Boxes indicate the interquartile range with the median line; whiskers denote minimum and maximum. Significance levels: ns = not significant; *p < 0.05; **p < 0.01; ***p < 0.001;  ****p < 0.0001

When analyzing circulating blood cells, we observed a significant increase in total leukocytes driven predominantly by neutrophils, whereas lymphocytes transiently increased at 6 km but decreased again following reoxygenation (Fig. 1). Monocyte counts remained stable across all time points. Hematocrit, mean corpuscular volume (MCV), and red-blood-cell distribution width (RDW) decreased slightly from baseline to the end of the experiment, while the number of erythrocytes and the mean corpuscular hemoglobin (MCH) content remained constant. Platelet counts showed a marked increase under hypoxia and a modest, non-significant decline after reoxygenation. Creatinine and creatine kinase (CK) levels decreased significantly at 6 km and remained lower after the experiment was completed, suggesting a reversible systemic adaptation rather than tissue injury.

### Complement profiling by targeted plasma proteomics

**Fig. 2:**
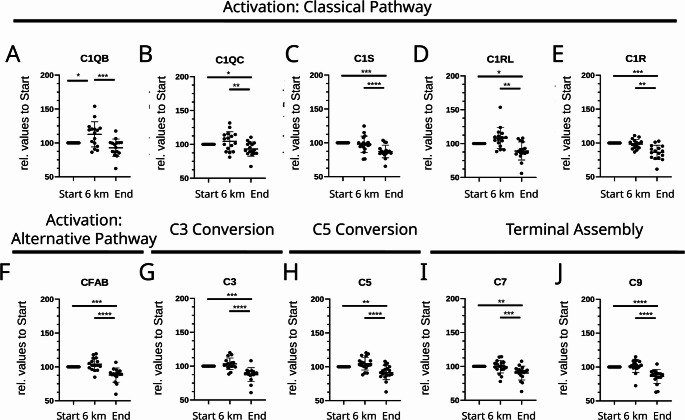
Quantitative mass-spectrometry analysis of complement system components. Relative peptide abundances of complement proteins were determined by using targeted multiple-reaction-monitoring (MRM) mass spectrometry and normalized to baseline values. Boxplots display relative abundances of C1QB, C1QC, C1R, C1RL, C1S, CFAB, C3, C5, C7, and C9 at baseline (Start), hypoxia (6 km), and reoxygenation (End). Proteins are grouped according to their activation pathway: classical (A-E), alternative (F), C3 conversion (G), C5 conversion (H), and terminal assembly (I-J). Single data points represent individual subjects (*n* = 16); Whiskers indicate standard deviation. Significance levels: *p < 0.05; **p < 0.01; ***p < 0.001; ****p < 0.0001

Given the large number of complement-associated proteins, peptide-based targeted mass spectrometry was employed to quantify representative components across the classical, alternative, and terminal pathways. These included the C1 complex peptides (C1S, C1R, C1RL, C1QB, C1QC), complement cascade intermediates (C3, C5, C7, C9), and complement factor B (CFAB) (Fig. 2; Table 1). During hypoxia, the relative peptide abundances remained close to baseline, indicating stable complement levels under reduced oxygen tension. In contrast, after reoxygenation, all analyzed peptides (i.e. C1S, C1R, C1RL, C1QB, C1QC, C3, C5, C7, C9, and CFAB) showed a consistent and significant decrease compared to both baseline and hypoxic conditions. This pattern shows a coordinated decrease in complement peptide abundance after reoxygenation, indicating oxygen-phase–dependent dynamics of the circulating complement pool. Because complement activation involves rapid proteolytic processing and consumption, reduced peptide abundance after reoxygenation is compatible with activation-associated consumption rather than suppression.

### Correlation analysis

**Fig. 3:**
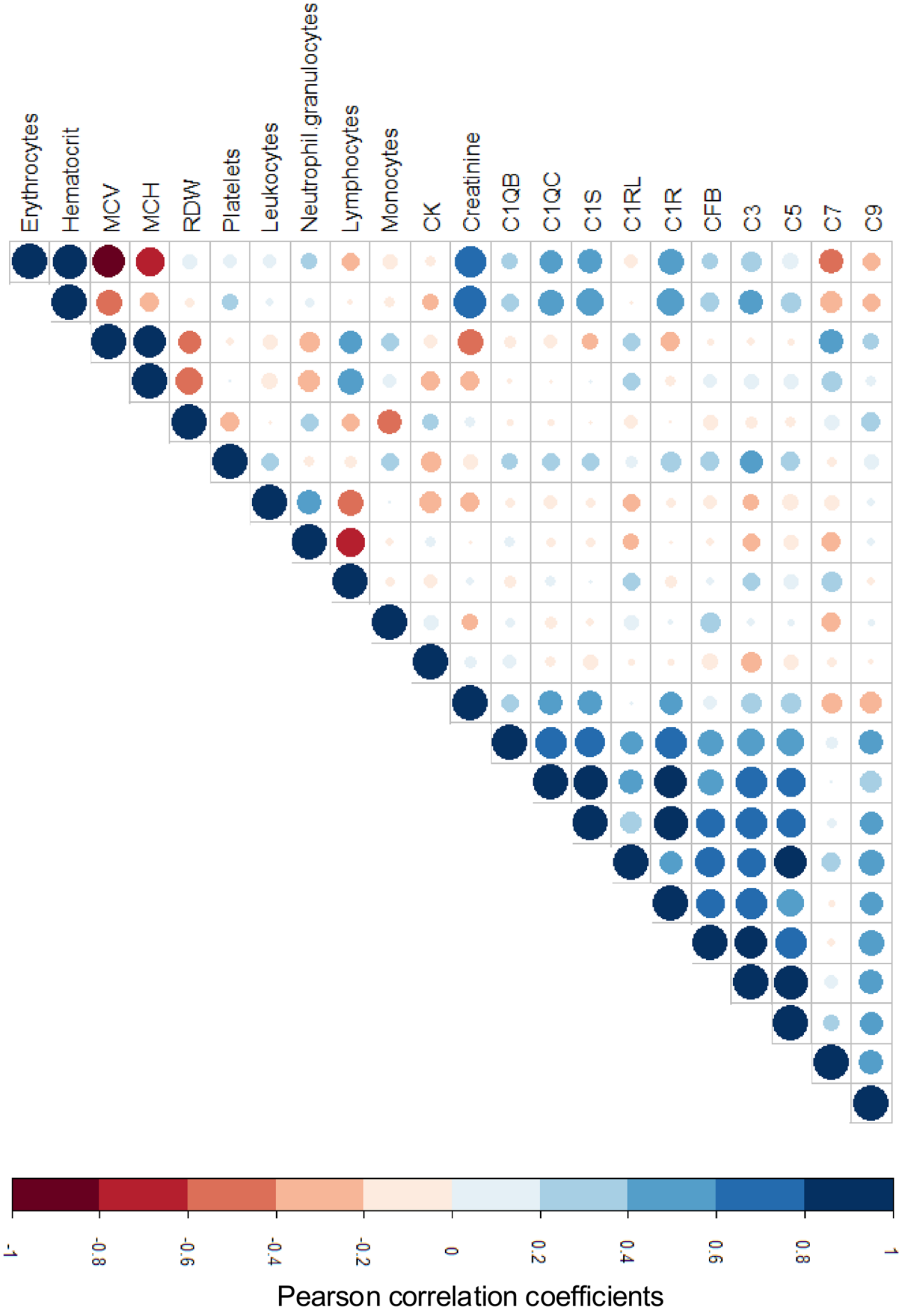
Correlation matrix of hematological and complement parameters. Heatmap showing correlations between 12 hematological parameters and quantified complement peptides (C1QB, C1QC, C1S, C1RL, C1R, CFB, C3, C5, C7, C9). Positive correlations are shown in blue and negative correlations in red. Circle size corresponds to the absolute correlation coefficient (|r|). CK = creatine kinase; HCT = hematocrit; MCV = mean corpuscular volume; MCH = mean corpuscular hemoglobin; RDW = red cell distribution width

To assess relationships between hematological variables and complement components, correlation matrices were generated (Fig. 3). Among hematological parameters, erythrocyte counts and hematocrit exhibited a strong positive correlation (*r* = 0.90), as did creatinine and hematocrit (*r* = 0.79), whereas erythrocytes and MCV displayed a pronounced negative correlation (*r* = − 0.83). Neutrophils and lymphocytes were inversely correlated (*r* = − 0.64), suggesting compensatory regulation within circulating leukocyte populations. RDW and MCH were negatively correlated (*r* = − 0.59), indicating that increasing size heterogeneity of erythrocytes coincided with lower hemoglobin content.

Within the complement network, strong positive correlations were detected among related components, particularly C1QB and C1QC (*r* = 0.73), C1QC and C1S (*r* = 0.86), C1S and C1R (*r* = 0.91), and C1RL and CFB (*r* = 0.67), consistent with their shared pathway architecture. Conversely, complement proteins showed only weak correlations with hematological indices, suggesting that complement modulation after hypoxia and reoxygenation is largely independent of changes in red or white blood cell profiles. Moderate associations were observed between C3 and hemoglobin (*r* = 0.40), C7 and MCH (*r* = 0.30), and C7 and MCV (*r* = 0.36), implying possible links between oxygen-carrying capacity and complement regulation. C9 displayed a moderate positive correlation with RDW (*r* = 0.31). All other cross-correlations were weak and not statistically significant.

## Discussion

The study demonstrates that acute, controlled hypoxia followed by immediate reoxygenation produces a reproducible and coordinated decrease in multiple circulating complement components in healthy humans. Complement levels remained stable during reduced oxygen tension but declined rapidly once normoxia was restored, demonstrating a reproducible oxygen-phase–dependent shift in the circulating complement peptide pool. The controlled design isolates oxygen dynamics from overt inflammation or tissue injury.

Complement is essential for host defense, immune complex clearance, and regulation of sterile inflammation [5]. Earlier studies have shown that complement activity contributes to tissue injury in ischemic and inflammatory settings, and that complement deficiency or blockade can reduce immune-mediated damage and improve tissue preservation in experimental models [14–16]. Under homeostatic conditions, regulatory proteins such as CD55 and CD59 prevent excessive activation by inhibiting C3 and C5 convertase formation and limiting membrane attack complex assembly [17, 18]. In conditions with reduced perfusion, tissue level hypoxia triggers cellular stress and inflammatory signaling [2]. In contrast, the coordinated reduction of complement components after reoxygenation observed in our study supports a rapid shift in the circulating complement pool during oxygen availability states.

Oxygen therapy, which aims to restore adequate respiratory metabolism in tissues after hypoxia, has the potential to mitigate complement-driven inflammation in diseases characterized by low perfusion [19]. These data define oxygen-phase–dependent complement dynamics under controlled physiological conditions and provide a baseline framework for disease-focused studies [20]. Whether oxygen restoration modulates complement activation in clinical hypoxemia requires dedicated functional assays and patient-cohort validation. Our data define oxygen-phase–dependent complement dynamics under controlled physiological conditions and provide a baseline framework for disease-focused studies. Furthermore, the hematological results obtained in the present study suggest that reoxygenation influences immune cell composition and erythrocyte morphology, consistent with earlier observations linking hypoxia to altered cell adhesion and RDW [21–23]. It is important to note, however, that oxygen therapy must be carefully managed, as rapid restoration of oxygen levels can trigger reperfusion injury through an abrupt rise in reactive oxygen species [24]. Therapeutic strategies should therefore emphasize controlled reoxygenation to minimize the risk of oxidative injury while maintaining immune modulation.

Clinical hypoxemia in disease states differs fundamentally from controlled normobaric hypoxia in healthy volunteers, because it typically co-occurs with endothelial injury, cytokine-driven inflammation, and tissue damage, all of which strongly shape complement activation. This is particularly evident in severe COVID-19, where hypoxemia is embedded in thrombo-inflammation and systemic immune dysregulation. Transcriptomic network analyses of SARS-CoV-2–infected bronchial epithelial cells integrating WGCNA with explainable machine learning (LIME) further highlight compartment-specific immune regulation that may not be captured by plasma proteomics alone [25]. 

Our data support oxygen-phase–dependent complement dynamics across hypoxia and reoxygenation. Hypoxia has been linked to changes in complement expression and activity in several settings, including tumors. Similar up-regulation of complement proteins was described in the hypoxic tumor microenvironment [11, 26]. When oxygen levels are restored, however, complement proteins decline rapidly, possibly reflecting consumption through activation or the restoration of inhibitory pathways under normoxic conditions.

Our findings link oxygen transitions to measurable changes in the circulating complement peptide pool, a central arm of innate immunity. However, several limitations must be considered. The study involved a limited number of healthy volunteers, and the results cannot be directly extrapolated to patients with chronic hypoxia or cardiovascular disease. Disease-associated hypoxemia combines hypoxia with inflammation and tissue injury; this study isolates the oxygen component. Furthermore, only a single acute normobaric hypoxia–reoxygenation cycle was analyzed, limiting temporal resolution. The underlying molecular mechanisms were not investigated in detail, and functional complement activation markers (C3a, C5a, membrane attack complex) as well as key regulators (e.g., factor H, factor I, C1 inhibitor) were not assessed. In this setting, a coordinated decrease in complement peptides can reflect a shift in the circulating complement pool driven by activation/consumption and redistribution. Accordingly, changes in complement peptide abundance cannot be interpreted as direct measures of complement functional activity. In addition, hypoxia and reoxygenation can alter plasma volume through fluid shifts and vascular tone regulation, that may contribute to apparent changes in circulating protein concentrations.

This study provides quantitative evidence that acute, reversible hypoxia followed by reoxygenation is accompanied by coordinated changes in circulating complement peptides in humans. The decrease after reoxygenation defines a consistent oxygen-phase signature and motivates follow-up studies in hypoxia-associated disease settings with functional complement readouts. These findings position oxygen transitions as a robust determinant of the circulating complement peptide signature. Future work should test whether similar dynamics occur in clinical hypoxemia and ischemia–reperfusion settings using functional complement activation markers.

## Data Availability

The data underlying this article are available in the Zenodo repository at https://doi.org/10.5281/zenodo.7422265.

## Abbreviations

pO2: Partial pressure of oxygen
pCO2: Partial pressure of carbon dioxide
CK: Creatine kinase
MCH: Mean level of corpuscular hemoglobin
MCV: Mean corpuscular volume
RDW: Red blood cell distribution width
RBC: Red blood cell
WBC: White blood cell
SpO2: Peripheral oxygen saturation
CFAB: Complement factor B
C1QB: Complement C1q subcomponent subunit B
C1QC: Complement C1q subcomponent subunit C
C1R: Complement C1r subcomponent
C1RL: Complement C1r subcomponent-like protein
C1S: Complement C1s subcomponent
C3: Complement C3
C5: Complement C5
C7: Complement component C7
C9: Complement component C9
HCT: Hematocrit
MS: Mass spectrometry
BMI: Body mass index
